# PACAP neuropeptide promotes Hepatocellular Protection via CREB-KLF4 dependent autophagy in mouse liver Ischemia Reperfusion Injury

**DOI:** 10.7150/thno.42354

**Published:** 2020-03-15

**Authors:** Zhengze Xue, Yu Zhang, Yuanxing Liu, Cheng Zhang, Xiu-da Shen, Feng Gao, Ronald W. Busuttil, Shusen Zheng, Jerzy W. Kupiec-Weglinski, Haofeng Ji

**Affiliations:** 1 Department of Surgery, Division of Hepatobiliary and Pancreatic Surgery, The First Affiliated Hospital, Zhejiang University, School of Medicine, Hangzhou, Zhejiang, China.; 2 Dumont-UCLA Transplant Center, Division of Liver and Pancreas Transplantation, Department of Surgery, David Geffen School of Medicine at University of California-Los Angeles, Los Angeles, CA, USA.

**Keywords:** Liver Ischemia Reperfusion Injury, Orthotopic Liver Transplantation, PACAP, KLF4, CREB

## Abstract

Organ ischemia reperfusion injury (IRI), associated with acute hepatocyte death, remains an unresolved problem in clinical orthotopic liver transplantation (OLT). Autophagy, an intracellular self-digesting progress, is responsible for cell reprograming required to regain post-stress homeostasis.

**Methods**: Here, we analyzed the cytoprotective mechanism of pituitary adenylate cyclase-activating polypeptide (PACAP)-promoted hepatocellular autophagy in a clinically relevant mouse model of extended hepatic cold storage (4 °C UW solution for 20 h) followed by syngeneic OLT.

**Results**: In contrast to 41.7% of liver graft failure by day 7 post-transplant in control group, PACAP treatment significantly improved graft survival (91.7% by day 14), and promoted autophagy-associated regeneration programs in OLT. In parallel *in vitro* studies, PACAP-enhanced autophagy ameliorated cellular damage (LDH/ALT levels), and diminished necrosis in H_2_O_2_-stressed primary hepatocytes. Interestingly, PACAP not only induced nuclear cAMP response element-binding protein (CREB), but also triggered reprogramming factor Kruppel-like factor 4 (KLF4) expression in IR-stressed OLT. Indeed, CREB inhibition attenuated hepatic autophagy and recreated hepatocellular injury in otherwise PACAP-protected livers. Furthermore, CREB inhibition suppressed PACAP-induced KLF4 expression, whereas KLF4 blockade abolished PACAP-promoted autophagy and neutralized PACAP-mediated hepatoprotection both *in vivo* and *in vitro*.

**Conclusion**: Current study documents the essential neural regulation of PACAP-promoted autophagy in hepatocellular homeostasis in OLT, which provides the emerging therapeutic principle to combat hepatic IRI in OLT.

## Introduction

Liver ischemia reperfusion injury (IRI), an innate immunity-mediated sterile inflammation, represents a major complication in liver transplantation, hemorrhagic shock and partial hepatectomy 1. It may lead to primary graft dysfunction and acute rejection episodes, which further deteriorate the organ shortage 1. Liver IRI disturbs local blood circulation, generates reactive oxygen species (ROS), assembles peripheral neutrophils/macrophages, and inflicts tissue damage resulting in ultimate organ failure. Our group was the first to demonstrate that neuropeptide PACAP, which innervates the hepatic cells, suppressed liver innate immune activation in a mouse model of liver warm ischemia followed by reperfusion 2,3. The current study examined whether and how PACAP promoted hepatocellular autophagy to restore hepatocellular homeostasis in a clinically authentic model of hepatic cold IRI-OLT.

Autophagy, an intracellular self-digesting progress degrading damaged organelles and malformed proteins by lysosomes, provides essential supply (amino acids, lipids and free fatty acid) to starved cells for energy metabolism and biosynthesis 4. As various cargo transports to the lysosome, three autophagy forms have been identified, i.e., macroautophagy, microautophagy, and chaperone-mediated autophagy. Macroautophagy, the main autophagic pathway, could be divided into six principle steps to form the autophagosome: initiation, nucleation, elongation, closure, maturation and degradation or extrusion 5,6. Fusing with lysosome, the autophagosome turns to autolysosome, in which the enclosed cytoplasmic materials are eventually degraded into active monomers 5,6. As liver is rich in lysosome, dysregulation of macroautophagy (hereafter referred to as autophagy) is associated with multiple hepatic diseases, i.e., IRI, non-alcoholic steatohepatitis and liver cancer 7-9. In liver transplantation, both warm and cold ischemia insults mainly interrupt the blood flow/oxygen supply, deplete hepatocellular adenosine triphosphate and produce ROS. This hepatocellular starvation significantly triggers autophagy signaling, which subsequently provides reused monomers to maintain cellular homeostasis 5,6,7,10.

Although liver IRI is considered as a local sterile innate response, both immune and nervous systems are integrated to maintain dynamic communication. IR-triggered inflammation was shown to activate systemic neuroendocrine hypothalamus and regional neural-hormonal-stress responses 11-13. Neural regulation optimizes, monitors and adjusts immune response, as a pivotal feedback loop by stimulation of efferent vagus nerve activity 11,12. After elimination of invasion, the neural system modulates local immune response towards host homeostasis 13.

PACAP, encoded by the ADCYAP1 gene, is a member of vasoactive intestinal peptide (VIP)/glucagon/secretin family 14. PACAP, contains two bioactive forms as 27 or 38 amino acid residues, functions as a neurotransmitter and neuromodulator in hypophysiotropic area, and involves in paracrine and autocrine regulation of gonadotrophs 15,16. Consistent with others' finding that PACAP exhibits neuroregulation on innate immunity 17, we have reported that partial warm IR triggered the inductions of hepatic PACAP and all three receptors in IR-stressed liver, whereas PACAP deficiency sensitized liver against IR-stress. Indeed, exogenous PACAP neuropeptide regulated the local innate immunity by diminishing neutrophil/macrophage sequestration in IR-liver and differentially modulating pro-inflammatory programs 2.

CREB, binding to cAMP response elements, is a critical cellular transcription factor in neuroregulation and neuron survival 18-20. In innate immunity, CREB ameliorates TLR4 response by regulating macrophage polarization in infection and insulin resistance 21,22. Recently, CREB was identified as an important regulator in hepatic autophagy gene network, promoting autophagic degradation of lipids 23. We have recently shown that PACAP inhibited macrophage NF-κB activity by enhancing cAMP-PKA axis and CREB activity 2,3.

KLF4, the crucial self-renewal transcriptional factor of KLF family, exerts diverse modulation in proliferation, differentiation, and somatic cell reprogramming 24. In addition to its crucial function in pluripotent stem cell induction 25,26, KLF4 is over-expressed in DNA-damaged non-dividing cells, and significant in inducing cell cycle arrest/preventing cell division 27,28. Indeed, KLF4 regulates macrophage polarization by cooperating with Stat6 to induce M2 gene profile and sequestering NF-κB activators to suppress M1 phenotype 29. Conversely, KLF4 extends the lifespan in nematode and modulates aged vascular dysfunction in mice by promoting autophagy 30. Putative synergy between KLF4 and CREB in modulation of innate immunity was discovered in mycobacterium tuberculosis 21.

This study elucidated the cytoprotective role of PACAP-mediated autophagy in a clinically authentic mouse model of extended hepatic cold storage and followed syngeneic OLT. First, we determined whether and how PACAP neuropeptide protected liver grafts against extended cold storage in an autophagy-dependent manner. The question then arose whether PACAP-mediated autophagy could preserve hepatocyte viability, and promote hepatic regeneration/repair. Finally, we addressed whether PACAP-mediated CREB and KLF4 coactivation was essential for hepatic autophagy in liver IRI.

## Materials and Methods

### Liver extended cold storage followed by syngeneic OLT model

Liver grafts from C57BL/6 mice were stored in 4°C UW solution for 20 h, and then transplanted orthotopically into syngeneic C57BL/6 mice (male, 8-12 weeks old, Jax lab, Bar Harbor, ME) 31-34. Surgical details: after the recipient liver was removed, donor suprahepatic vena cava was anastomosed end to end with recipient suprahepatic vena cava; both portal vein and infrahepatic vena cava were reconnected through a cuff technique; and bile duct reconstruction was completed with an intraluminal stent. Groups of recipients were given PBS, PACAP38 (1 mg/kg via portal vein, dissolved in PBS, Phoenix Pharmaceuticals, Burlingame, CA), supplement of Rapamycin (3 mg/kg via portal vein, MilliporeSigma, Burlington, MA) or 3-Methyladenine (3-MA) (100 μg/kg via portal vein, dissolved in DMSO, MilliporeSigma) at the time of liver graft procurement and immediately prior to reperfusion. Because all inhibitors (3-MA, CREB inhibitor and KLF4 inhibitor) were dissolved in DMSO, we added DMSO in PACAP solution for functional control. OLT survival was observed and the serum and OLT samples were collected at 6 h and 24 h post-transplant.

### Partial liver warm IRI model

The artery and portal vein of cephalad lobes (70% of whole liver) were clamped by an atraumatic clip for 90 min 2,3,35-37. A single dose of PACAP38 (1 mg/kg i.v., dissolved in PBS) or CRP-CREB Interaction Inhibitor (CREB inhibitor: CAS92-78-4, 250 μg/kg i.v., dissolved in DMSO, MilliporeSigma), or KLF4 inhibitor (Kenpaullone, 250 μg/kg i.v., dissolved in DMSO, MilliporeSigma 38) was given 1 h before warm ischemia. Ischemic liver tissue and serum samples were harvested at 6 h post reperfusion. All mice surgeries were approved by UCLA Animal Research Committee.

### Liver function and histology

Serum alanine transaminase (sALT) indicated liver function was assessed by IDEXX Lab (Sacramento, CA). Hematoxylin and eosin-stained liver sections and electron microscopy imagine were analyzed blindly for liver histology.

### Quantitative RT-PCR

Quantitative PCR was performed by using Platinum SYBR green quantitative PCR kit and QuantStudio3 Real-time PCR system (ThermoFisher). The used primer sequences were published 31-37 or shown in Table S1: LC3a, LC3b, Beclin-1, EGF, HGF, c-Met, KLF1, KLF2, KLF3 and KLF4. The ratios of target gene induction to housekeeping gene HPRT were calculated.

### Western blots

Ischemic liver tissue proteins, after electrophoretic separation and transfer, were detected by monoclonal antibodies of Atg5 (12944), LC3 (4108), p62 (39749), proliferating cell nuclear antigen (PCNA) (13110), Ki67 (9129), pCREB (9198), KLF4 (12173), and β-actin (4970) (Cell Signaling Technology, Danvers, MA).

### H_2_O_2_ stressed hepatocytes assay

After liver collagenase perfused digestion* in situ,* primary hepatocytes were separated by Percoll gradient centrifugation, and then cultured in collagen-coated plate for 24 h. Pre-treatment with PACAP38 (10 nM), with 3-MA (10 μM), CREB inhibitor (10 μM), KLF4 inhibitor (10 μM), DMSO for 1 h, KLF4 siRNA or NC siRNA (transfected by Lipofectamine 2000, ThermoFisher), hepatocellular necrosis was triggered by hydrogen peroxide (H2O2: 0.4 mM, MilliporeSigma). Cells were processed for immunofluorescence and PI staining after 6 h, whereas supernatants were assessed by ALT/LDH kit (ThermoFisher).

### Immunofluorescence staining

Stressed hepatocytes were stained with LC3 (4108), pCREB (9198) and KLF4 (12173) monoclonal antibodies (Cell Signaling Technology) followed with Alexa Fluor 488 secondary antibody (A11008), Alexa Fluor 555 F-actin conjugate (A34055) for cellular skeleton, and DAPI counterstain (P36931) (ThermoFisher).

### Propidium Iodide (PI) staining

PI staining (ThermoFisher) was used to detect dead cells by binding DNA. Red fluorescence images were merged with bright-field images for blindly evaluation.

### Statistical analysis

Liver graft survival curves were plotted by Kaplan-Meier survival analysis and Log-rank comparison test. All results were indicated by mean ± standard deviation, and analyzed with Student's t test and ANOVA test. Statistical significant was identified as P<0.05.

## Results

### PACAP attenuates hepatic cold IRI and promotes OLT survival

**Figure 1:**
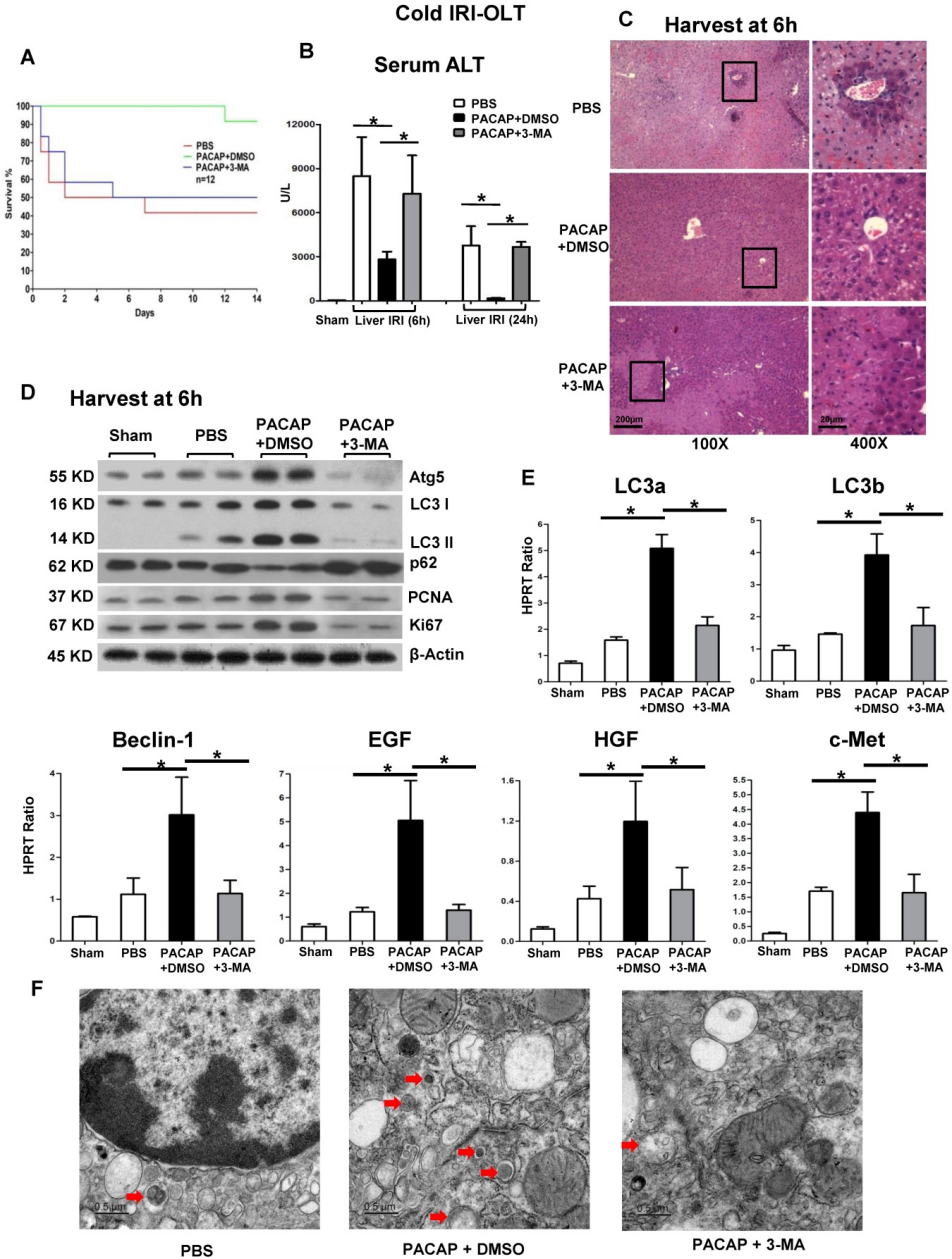
In syngeneic orthotopic liver transplantation subjected to extended cold storage (in 4°C UW solution for 20 h), there were three experimental groups (n = 12/group) of recipients treated with PBS, PACAP+DMSO or PACAP+3-MA at liver harvest and immediately prior to reperfusion through portal vein injection. **(A)** OLT survival rate was monitored during 14 days post-OLT: PBS (41.7%, red); PACAP+DMSO (91.7%, green, p<0.001); and PACAP+3-MA (50%, blue). Recipients were sacrificed at 6 h and 24 h post-transplant and OLT/serum samples were collected. **(B)** sALT levels; **(C)** liver pathology (H&E staining; magnification: x100: scale bar represents 200µm; x400: scale bar represents 20µm); **(D)** western blot of Atg5, LC3 I, LC3 II p62, PCNA, and Ki67; **(E)** qPCR-assisted detection of LC3a, LC3b, Beclin-1, EGF, HGF, and c-Met; **(F)** electron microscopy imagine of graft at 6 h post of OLT (red arrow indicated autophagosome, scale bar represents 0.5µm) (*p<0.001, n=4-6/group).

First, we studied whether administration of PACAP neuropeptide protected liver against extended cold storage-mediated IRI and prolonged graft survival in a mouse syngeneic OLT model. Compared with 41.7% survival in PBS control group, 91.7% of recipients conditioned with PACAP remained alive at 14 days post-OLT (Figure 1A, p<0.001). As cold IR-exacerbated hepatocellular damage peaks at 6 h post-reperfusion 31-34, we then assessed OLT and sera samples at 6 h and 24 h. Similar as Rapamycin treated group (Figure S1, autophagy agonist), PACAP treatment significantly diminished IRI-OLT, as shown by decreased sALT levels (Figure 1B, p<0.001) and preserved liver histology (without necrosis or congestion) (Figure 1C).

### PACAP promotes hepatic autophagy

PACAP therapy in IR-stressed livers enhanced hepatic autophagy induction, as assessed by expression of several key components (LC3, Beclin-1 and Atg5), as compared with controls (Figure 1D-E, p<0.001). Consistently, PACAP neuropeptide augmented the expression level of LC3, diminished p62 level, as well as promoted the LC3 I to LC3 II conversion, which was identified as the critical active step in autophagy pathway (Figure 1D). Electron micrographs of autophagosome (red arrow) in liver graft at 6 h post of OLT were shown in Figure 1F.

### PACAP-mediated cytoprotection/regeneration in OLT is autophagy-dependent

We then determined the significance of PACAP-mediated hepatic autophagy in liver homeostasis by using an autophagy inhibitor 3-MA, which blocks the autophagosome formation in IR-stressed OLT. Autophagy inhibition diminished the induction of LC3 I, LC3 II and Beclin-1 (Figure 1D-E) and restored cardinal IRI-OLT features, i.e., increased sALT levels (Figure 1B) and deteriorated hepatic architecture (Figure 1C). Of note, PACAP monotherapy-mediated autophagy amplified downstream hepatocellular regeneration, i.e., PCNA, Ki67, epidermal growth factor (EGF), hepatocyte growth factor (HGF) and their receptor c-Met, as compared with controls (Figure 1D-E, p<0.001); whereas autophagy inhibition suspended hepatocyte repairing programs (PCNA, Ki67, EGF, HGF and c-Met) induced otherwise by PACAP-mediated autophagy (Figure 1D-E, p<0.001).

### PACAP-mediated autophagy prevents hepatocyte death

**Figure 2:**
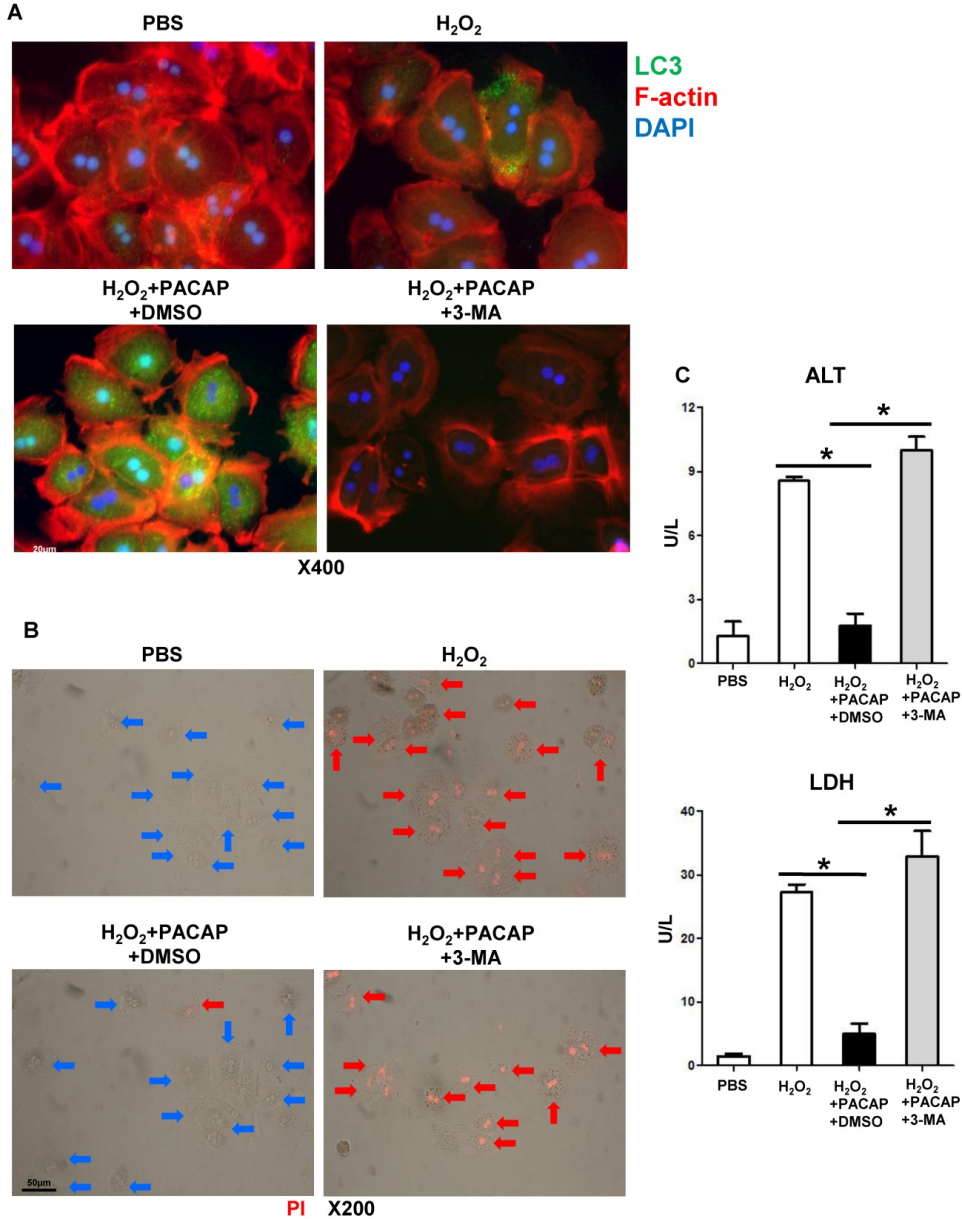
PACAP-mediated hepatocellular autophagy *in vitro*. Primary hepatocytes were pre-treated with PACAP+DMSO, or PACAP+3-MA 1 h prior to H_2_O_2_ stress. **(A)** Representative immunofluorescence staining: LC3 (green), cellular skeleton (F-actin, red) and nuclear (DAPI, blue): LC3 accumulation in cytoplasm and nucleus (magnification: x400, scale bar represents 20µm); **(B)** PI staining: red fluorescence images (PI stained nuclear of dead cells) were merged with brightfield images: live cells (blue arrows) and dead cells (red arrows) (magnification: x200, scale bar represents 50µm); **(C)** supernatant ALT/LDH levels (*p<0.001, n=6/group).

We then analyzed neural modulation of PACAP-mediated autophagy in a well-controlled primary hepatocyte culture stimulated by hydrogen peroxide (H_2_O_2_), a system designed to mimic liver IRI *in vivo*. PACAP pre-treatment preferentially enhanced LC3 induction and accumulation in hepatocellular nucleus and cytoplasm (Figure 2A: green fluorescence indicative of LC3), protected hepatocytes against oxidative stress (as shown by lower supernatant releasing of ALT/LDH (Figure 2C), and prevented hepatocyte death (Figure 2B: red fluorescence indicative of dead hepatocytes as PI cannot permeate live cells to stain nuclear chromosome). As 3-MA abolished LC3 accumulation in hepatocytes (Figure 2A: no green fluorescence), autophagy inhibition exacerbated hepatocellular damage, evidenced by higher ALT/LDH (Figure 2C, p<0.001) and abundant PI positive hepatocytes (Figure 2B).

### PACAP-CREB axis is critical for hepatic autophagy in liver IRI

**Figure 3:**
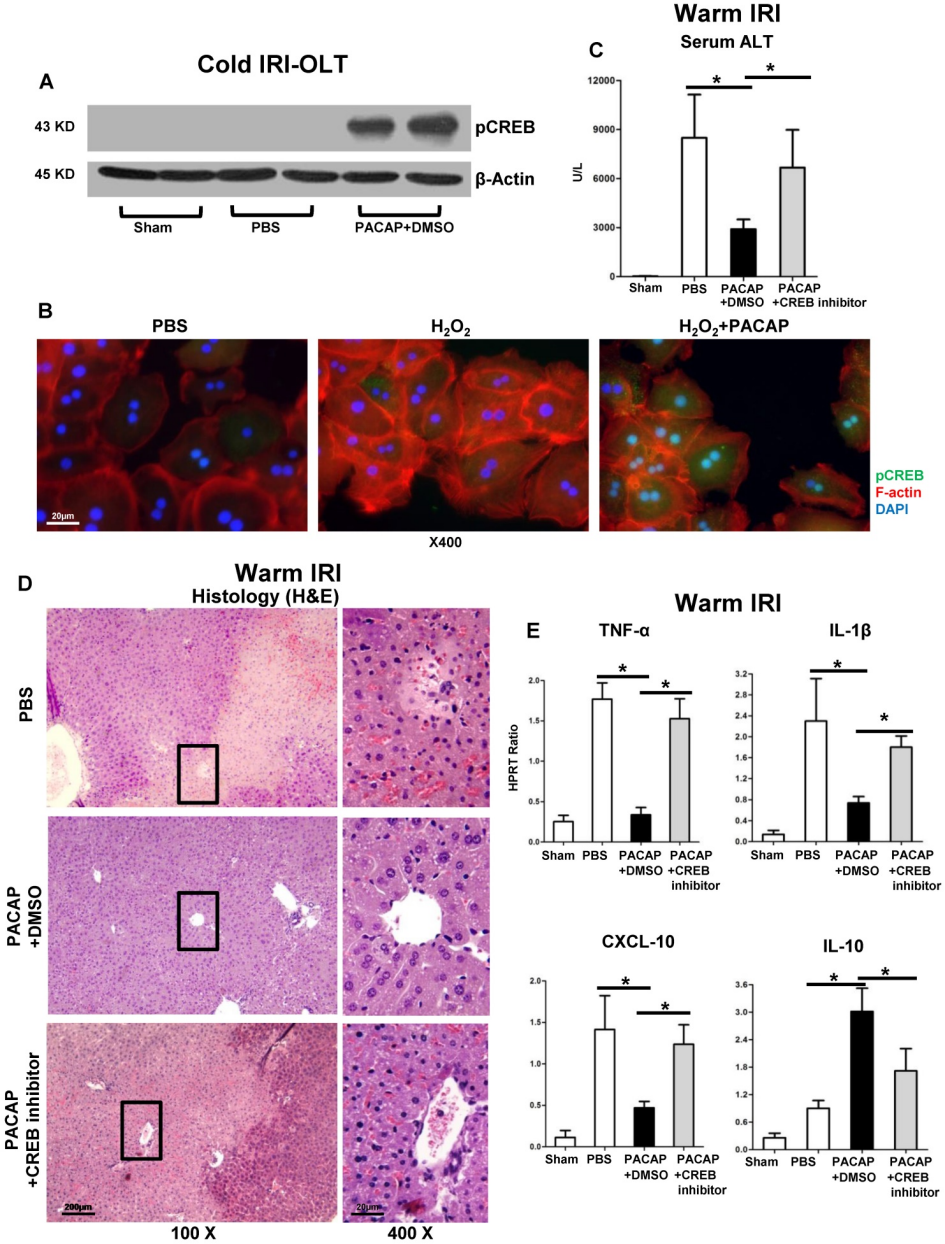
PACAP-CREB axis in liver IRI. **(A)** Western blot of pCREB in cold storage stressed OLT; **(B)** representative immunofluorescence staining of pCREB (green), F-actin (red) and DAPI (blue): pCREB accumulation in nuclear (magnification: x400, scale bar represents 20µm). PACAP+DMSO, PACAP+CREB inhibitor or PBS pre-treated WT mice were subjected to partial liver warm ischemia (90 min). Ischemic liver tissues were harvested at 6 h of reperfusion for **(C)** sALT levels; **(D)** liver pathology (H&E staining; magnification: x100: scale bar represents 200µm; x400: scale bar represents 20µm); (E) qPCR of TNF-α, IL-1β, CXCL-10 and IL-10 (*p<0.001, n=4-6/group).

As we have shown PACAP activated PKA-cAMP-CREB signaling in liver IRI 2,3, we consistently detected hepatic pCREB activation in cold-stored OLT (Figure 3A); and oxidative stressed hepatocyte cultures (Figure 3B: green fluorescence indicative of pCREB in hepatocellular nucleus/cytoplasm).

We then used a well-established murine model of partial liver warm ischemia (90 min) and reperfusion (6 h) to study the mechanism of PACAP-CREB axis-mediated autophagy in IR-damage. In agreement with our previous results 2,3, PACAP pre-treatment strongly depressed IR-mediated hepatocellular damage (Figure 3C-D); while CREB inhibition restored hepatic IR-damage, as shown by enhanced sALT levels and exacerbated IR-injured histology (Figure 3C-D). In parallel, CREB inhibition enhanced pro-inflammatory immune profile (Figure 3E: TNF-α, IL-1β and CXCL-10, p<0.001) while reducing IL-10 level (Figure 3E, p<0.001).

### CREB is required for PACAP-mediated hepatic autophagy in liver IRI

**Figure 4:**
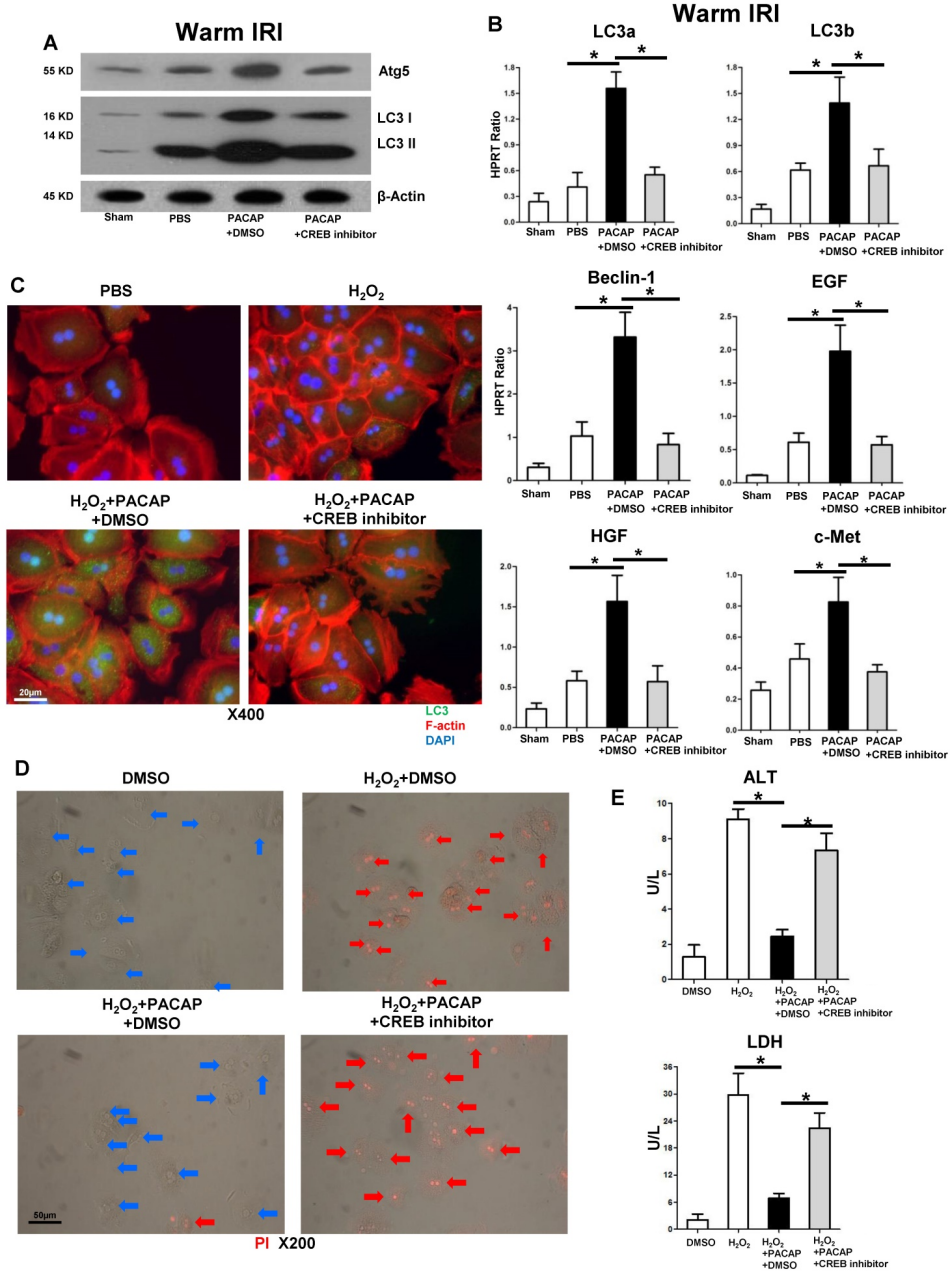
CREB activation is essential in PACAP-mediated hepatic autophagy in liver IRI. PACAP+DMSO, PACAP+CREB inhibitor or PBS pre-treated WT mice were subjected to partial liver warm ischemia (90 min). Ischemic liver tissues were harvested at 6 h of reperfusion for **(A)** western blot of Atg5, LC3 I and LC3 II; **(B)** qPCR-assisted detection of LC3a, LC3b, Beclin-1, EGF, HGF, and c-Met (n=4-6/group, *p<0.001). **(C)** Immunofluorescence staining: LC3 (green), F-actin (red) and DAPI (blue): LC3 accumulation in cytoplasm and nuclear (magnification: x400, scale bar represents 20µm); **(D)** PI staining (red): live cells (blue arrows) and dead cells (red arrows) (magnification: x200, scale bar represents 50µm); **(E)** supernatant ALT and LDH levels (*p<0.001, n=6/group).

As PACAP promoted hepatic autophagy in liver IRI, PACAP administration failed to activate autophagy-related gene expression program after CREB inhibition *in vivo* (Figure 4A, p<0.001) and in oxidative stressed hepatocyte cultures (Figure 4C), implying that PACAP mediated hepatocellular autophagy was CREB-dependent. Furthermore, CREB inhibition abolished PACAP-mediated hepatocellular regeneration (Figure 4B), recreated oxidation-mediated hepacellular death and ALT/LDH release in hepatocytes *in vitro* (Figure 4D-E).

### PACAP-CREB signaling controls KLF4 in liver IRI

**Figure 5:**
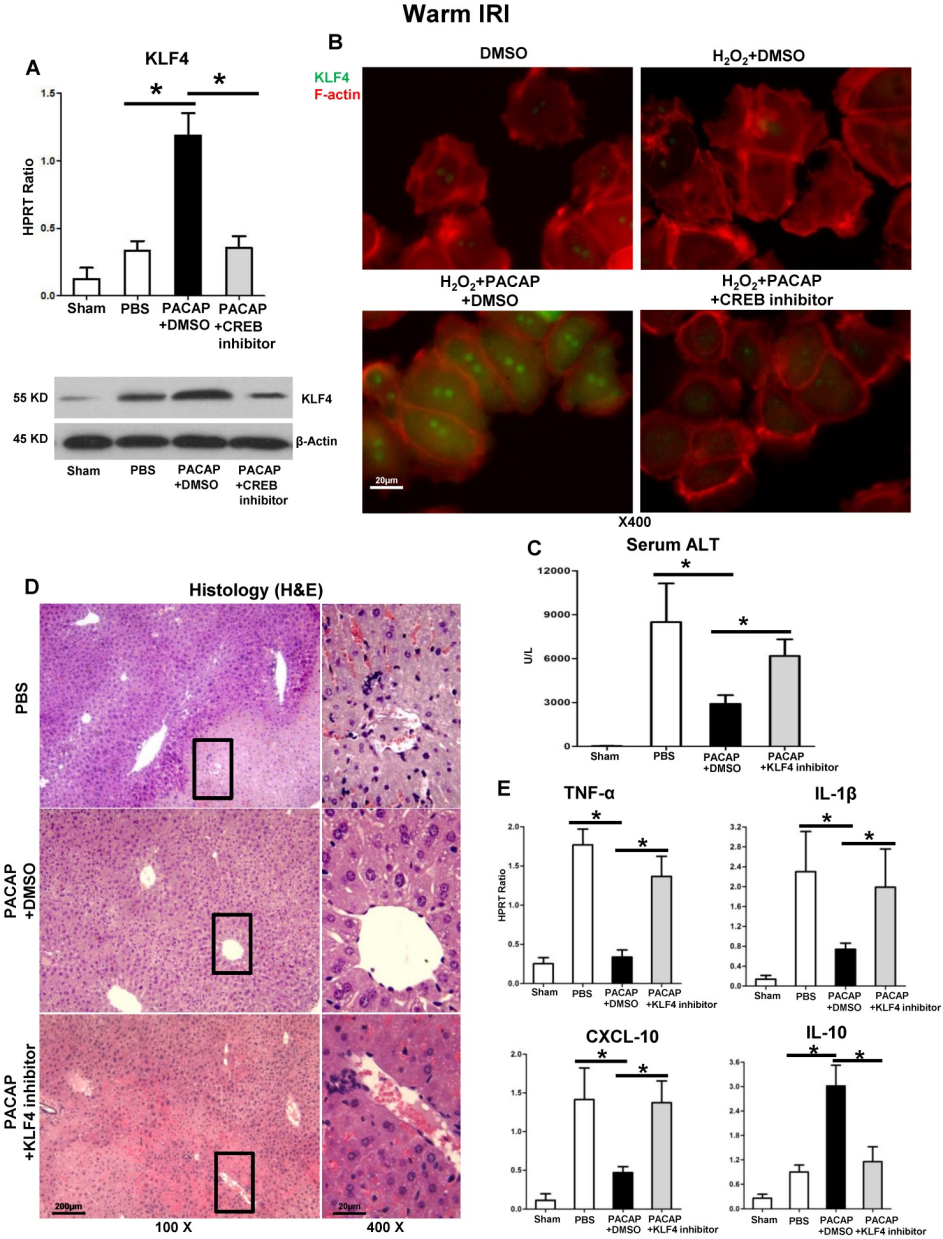
KLF4 in PACAP-CREB axis controls hepatocellular function in liver IRI. **(A)** qPCR and western blot of KLF4 in ischemic livers (*p<0.001); **(B)** Representative immunofluorescence staining of KLF4 (green) and F-actin (red): nuclear KLF4 accumulation (magnification: x400, scale bar represents 20µm). PACAP+DMSO, PACAP+KLF4 inhibitor or PBS pre-treated WT mice were subjected to partial liver warm ischemia (90 min). Ischemic liver tissues were harvested at 6 h of reperfusion for** (C)** sALT levels; **(D)** liver histology (representative H&E staining; magnification: x100: scale bar represents 200µm; x400: scale bar represents 20μm); **(E)** qPCR-assisted detection of TNF-α, IL-1β, CXCL-10 and IL-10 (*p<0.001, n=4-6/group).

Compared with controls, PACAP enhanced KLF4 gene induction (but not other KLFs (Figure S2)) and protein expression in both liver IRI (Figure 5A) and* in vitro* hepatocyte cultures (5B: green fluorescence indicative of KLF4 in hepatocellular nucleus/cytoplasm), which otherwise are diminished after CREB inhibition (Figure 5A-B). We then employed KLF4 inhibitor in liver warm IRI. In analogy with PACAP-CREB ablation, KLF4 inhibition blunted PACAP-mediated cytoprotection, and restored IR-induced hepatocellular injury, as shown by increased sALT levels (Figure 5C) and deteriorated liver pathology (Figure 5D). In addition, KLF4 inhibition recreated PACAP-suspended pro-inflammatory cytokine program (Figure 5E: TNF-α, IL-1β, CXCL-10, p<0.001), and suppressed IL-10 expression (Figure 5E, p<0.001).

### KLF4 is essential for PACAP-CREB-mediated hepatic autophagy in liver IRI

**Figure 6:**
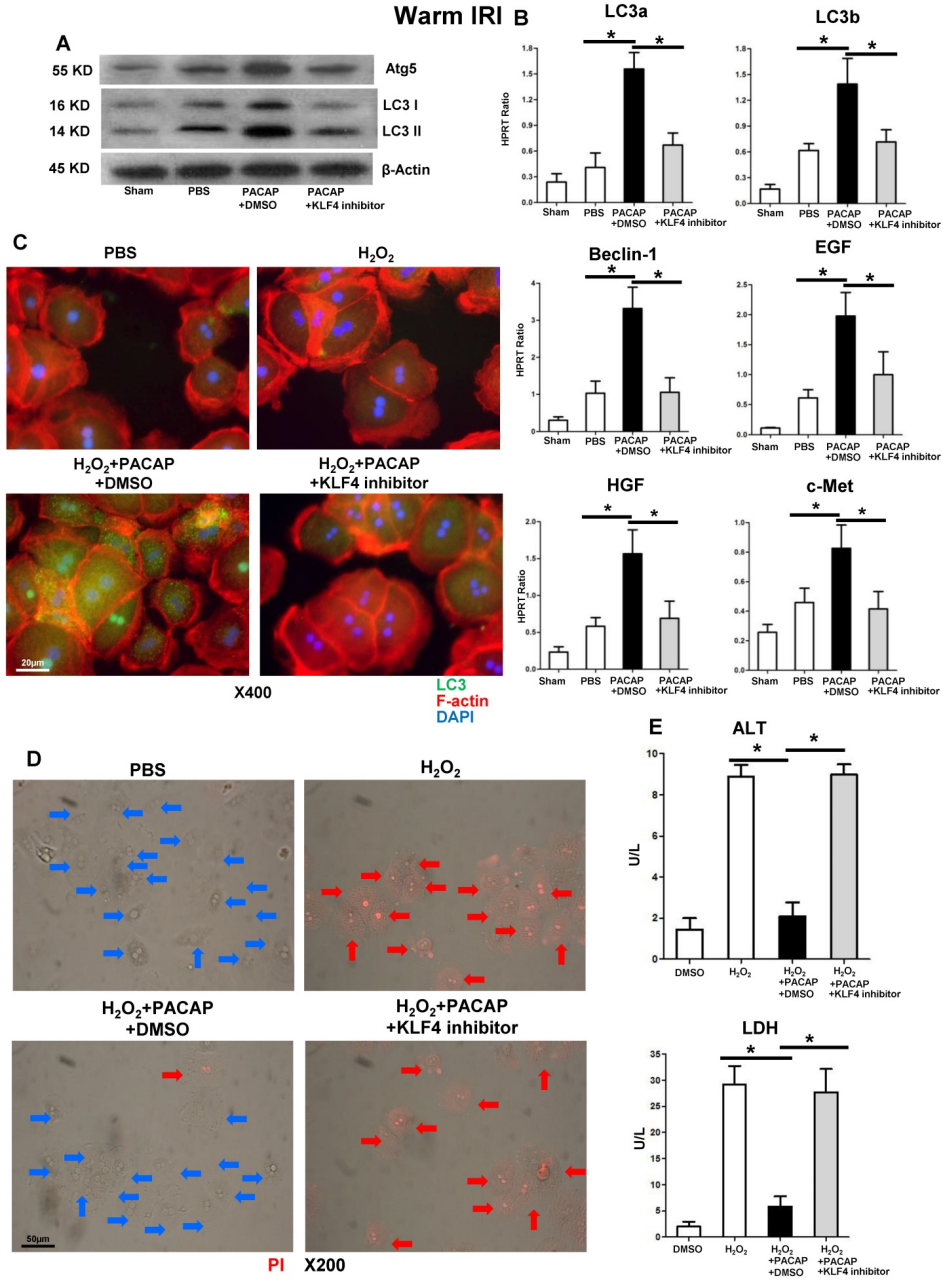
KLF4 is critical in PACAP-CREB axis-mediated hepatic autophagy in liver IRI. PACAP+DMSO, PACAP+KLF4 inhibitor or PBS pre-treated WT mice were subjected to 90 min ischemia. Liver samples were analyzed at 6 h of reperfusion for **(A)** western blot-assisted detection of Atg5, LC3 I and LC3 II; **(B)** qPCR-assisted detection of LC3a, LC3b, Beclin-1, EGF, HGF, and c-Met (n=4-6/group, *p<0.001). **(C)** Immunofluorescence staining: LC3 (green), F-actin (red) and DAPI (blue): LC3 accumulation in cytoplasm and nucleus (magnification: x400, scale bar represents 20µm); **(D)** PI staining (red): live cells (blue arrows) and dead cells (red arrows) (magnification: x200, scale bar represents 50µm); **(E)** supernatant ALT and LDH levels (*p<0.001, n=6/group).

KLF4 suspension significantly diminished otherwise abundant hepatic autophagy related components (Figure 6A) and hepatocellular regeneration programs (Figure 6B) in PACAP-conditioned liver IRI. Consistently, KLF4 inhibition attenuated PACAP-mediated hepatocellular autophagy in stressed hepatocyte cultures (Figure 6C), and restored hepatocyte damage, as shown by amplified cell death and ALT/LDH release (Figure 6D-E and Figure S3).

## Discussion

In a murine model of warm IRI, we have previously shown that treatment with PACAP neuropeptide controlled innate immunity-mediated macrophage function and modulated hepatic homeostasis 2,3. In the present study: (i) exogenous PACAP neuropeptide promoted liver graft survival in a mouse model of extended cold storage followed by OLT, which mimics the clinical scenario; (ii) PACAP-enhanced autophagy was essential for hepatic cytoprotection in IR-stressed OLT; (iii) PACAP-mediated hepatocellular regeneration in liver IRI was autophagy-dependent; and (iv) CREB-KLF4 axis was critical in PACAP-modulated liver autophagy and regaining homeostasis in IRI-OLT.

In a warm hepatic IRI model, PACAP treatment diminished macrophage inflammation response in PKA-dependent manner and prevented hepatocellular necrosis/apoptosis through YAP signaling 2,3. Others reported that PACAP induced DSCR1 39, which mediated mitochondrial autophagy in neurons 40. In our current cold ischemia-mediated OLT injury model, administration of PACAP neuropeptide ameliorated liver IRI by promoting autophagy program, i.e., hepatic expression of Atg5, LC3, Beclin-1 and transformation of LC3 I to LC3 II, the key elements for autophagosome formation; while enhancing the regenerative gene program (EGF, HGF, and c-Met), as compared with IRI-OLT controls. 3-MA inhibited autophagy by inhibition of class III Phosphatidylinositol 3-kinases (PI-3K), which is the activator of upstream of autophagy and CREB respectively 41. In contrast, administration of autophagy inhibitor 3-MA exacerbated hepatic IRI, abolished autophagy activation/liver regeneration, suggesting PACAP-mediated cytoprotection/hemostasis in cold IRI-OLT was an autophagy-dependent phenomenon. Inhibitors may have some limitations, i.e. multiple molecular targets, short life span and increasing the risk of toxicity. Indeed, we examined the hepatocellular autophagy in PACAP KO mice in a mouse model of extended cold storage followed by OLT. As the liver graft damage was exacerbated at 6 h post OLT, the hepatic autophagy was abolished in OLTs (data not shown).

To mimic liver IR-mediated hepatocellular hyperoxidation insult *in vivo,* we then analyzed PACAP modulation in a well-controlled hydrogen peroxide (H_2_O_2_) stimulated primary hepatocyte cultures. To further determine whether PACAP-exerted cytoprotection was autophagy-dependent, we first monitored the autophagy activity in PACAP treated primary hepatocytes by immunofluorescence staining. Markedly increased LC3 accumulation was found in hepatocellular nucleus and cytoplasm of PACAP-treated hepatocytes. Hyperoxidative hepatocellular necrosis is typically featured as loss of cell membrane integrity and releasing of cellular enzymes. As PI dye is unable to permeate through the intact cell membrane of live cells, PI stains dead cell DNA as diffusion into the porous membrane. Interestingly, PACAP prevented oxidation stressed hepatocellular death, while control group exhibited notable PI-indicated dead cells and hepatocellular release of ALT/LDH in supernatants. Consistently, autophagy inhibition eliminated LC3 accumulation in PACAP-treated hepatocytes, and recreated hyperoxidative cell death, evidenced by massive PI staining and elevated ALT/LDH levels.

Activation of CREB signaling preferentially promoted autophagy-mediated neuroprotection in a rat model of Alzheimer's disease 41. As the newly identified transcriptional activator of autophagy, CREB promoted lipophagy (autophagic degradation of intracellular lipid droplets) and activated total 112 autophagy related genes during nutrient deprivation 22,42. As previously discovered that PACAP-triggered PKA-CREB axis and CREB-YAP signaling in liver IRI 2,3,43,44, we then evaluated CREB phosphorylation in PACAP-enhanced hepatic autophagy. Having shown that PACAP neuropeptide significantly improved CREB activation, we next employed a CREB inhibitor to analyze PACAP-mediated modulation in IR-stressed liver. Strikingly, CREB antagonism not only potentiated hepatic pro-inflammation response in otherwise PACAP treated and IR-resistant hosts, but also recreated liver IR-damage. In parallel, both autophagy and regeneration-related gene expression programs, promoted by PACAP stimulation, were abolished by CREB inhibitor. Meanwhile, CREB inhibition impaired hepatocellular LC3 abundance in PACAP-promoted autophagy, and restored cellular death in oxidation-stressed hepatocyte culture. These findings support the contention that PACAP mediated PKA-CREB pathway is critical for hepatic autophagic cytoprotection in liver IRI.

KLF4, a pleiotropic transcription factor, regulates cellular differentiation, proliferation and oncogenesis 45,46. KLF4-mediated cell reprogramming contributed to tissue homeostasis in IR-damaged heart, brain and kidney 47-49, while KLF4 deficiency diminished autophagy in embryonic fibroblasts 50. It has been reported that CREB coactivated KLF4 transcription via histone acetylation 51. In fact, we observed PACAP neuropeptide promoted hepatic KLF4 expression in liver IRI, while CREB inhibitor abolished PACAP-mediated stimulation and eliminated KLF4 expression in primary hepatocytes. As KLF4 synergized with CREB in atherosclerosis and co-regulated the renal progenitor cell proliferation with PACAP 52, we then asked whether KLF4 contributed to the regulation of PACAP-CREB axis in our model. Indeed, KLF4 inhibition amplified macrophage innate immune activation, and impaired PACAP-CREB mediated autophagic cytoprotection. Further, KLF4 inhibitor abolished otherwise abundant LC3/Beclin-1 expression in PACAP-treated IR-stressed livers and oxidation-stressed primary hepatocytes, confirming the integrality of PACAP-CREB-KLF4 was indispensable in autophagy-mediated cytoprotection in liver IRI.

In conclusion, this study documented PACAP attenuated liver IR-injury and prolonged graft survival in a clinically relevant cold IR stressed OLT model. Exogenous PACAP neuropeptide-enhanced autophagy controls hepatic cytoprotection, while proficient PACAP-CREB-KLF4 signaling is essential for regaining hepatocellular homeostasis. Consistent with the emerging function of autophagy-mediated cytoprotection against IR-stress 53, this study supports PACAP neuropeptide as a novel treatment in liver transplantation.

## Supplementary Material

Supplementary figures and table.

## Abbreviations

3-MA: 3-Methyladenine
CREB: cAMP response element-bing protein
EGF: epidermal growth factor
HGF: hepatocyte growth factor
H_2_O_2_: hydrogen peroxide
IRI: ischemia/reperfusion injury
KLF4: Kruppel-like factor 4
OLT: orthotopic liver transplantation
PACAP: pituitary adenylate cyclase-activating polypeptide
PI: Propidium Iodide
ROS: reactive oxygen species
sALT: serum alanine transaminase
VIP: vasoactive intestinal peptide
