# Supplementary material for: PACAP neuropeptide promotes Hepatocellular Protection via CREB-KLF4 dependent autophagy in mouse liver Ischemia Reperfusion Injury
Source: Theranostics. 2020 Mar 15;10(10):4453–65. doi: 10.7150/thno.42354 (PMC7150481; doi:10.7150/thno.42354)
Supplement: Supplementary file 1 — Supplementary figures and table. [file thnov10p4453s1.pdf]

Figure S1

6h after Cold IRI-OLT

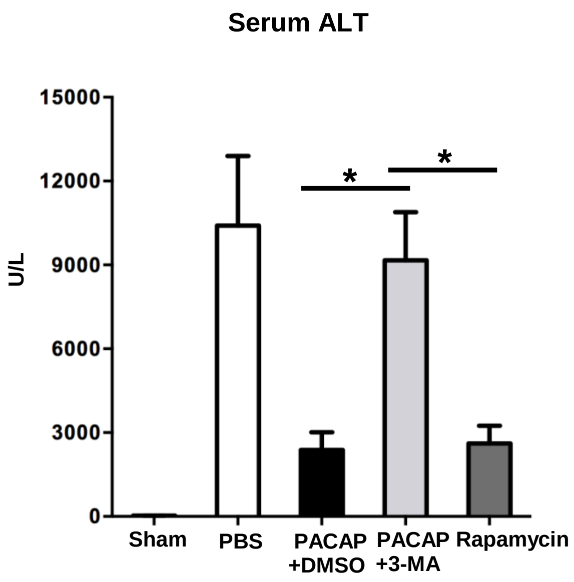

Figure S1 In syngeneic orthotopic liver transplantation subjected to extended cold storage (in 4° C UW solution for 20h), there were four experimental groups of recipients treated with PBS, PACAP+DMSO PACAP+3-MA or Rapamycin at liver harvest and immediately prior to reperfusion through portal vein injection. Serum ALT levels were analyzed at 6h post-transplant (*p<0.001, n=4-6/group).

Figure S2

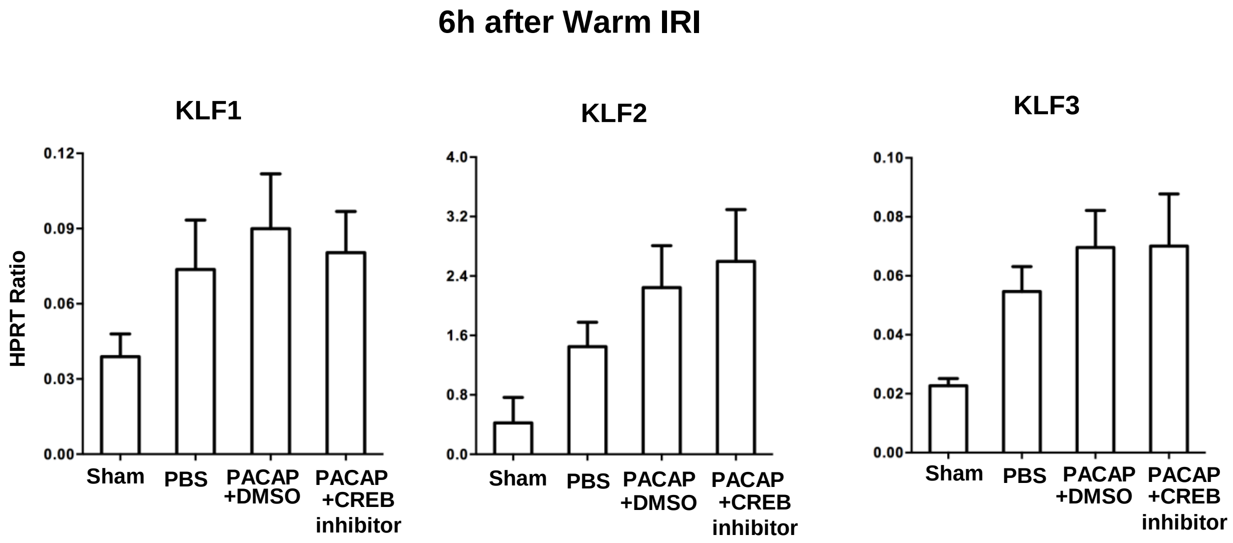

Figure S2 qPCR of KLF1, KLF2 and KLF3 in liver IRI (n=4-6/group).

Figure S3

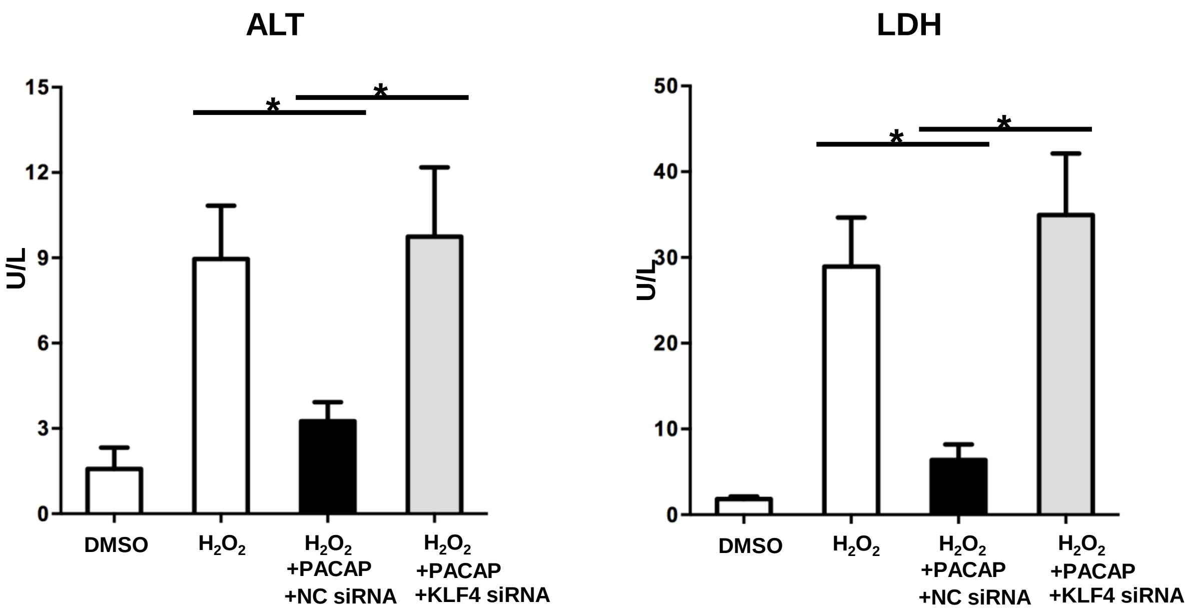

Figure S3 siRNA-mediated KLF4 silencing in vitro. Primary hepatocytes were pre-treated with PACAP+NC siRNA, or PACAP+KLF4 siRNA 1h prior to H2O2 stress. Supernatant ALT/LDH levels were assessed (*p<0.001, n=6/group).

Table S1

Primers used for qPCR

Genes	sense	antisense
LC3a	catgagcgagttggtcaaga	ccatgctgtgctggttga
LC3b	ctgaccacgtgaacatgagc	acttcggagatgggagtgg
Beclin-1	caggcgaaaccaggagag	cgagtttcaataaatggctcct
EGF	catgccccacaggatttg	gggcaggaaacaagttcg
HGF	caccccttgggagtattgtg	gggacatcagtctcattcacag
c-Met	ctgctctggaggacaagacc	gagttgatcacatgccaagc
KLF1	caagagctcgcacctcaag	gagcgaacctccagtcaca
KLF2	ctaaaggcgcatctgcgta	tagtggcgggtaagctcgt
KLF3	gtccagcccgttccttttat	agggcttctcatatgattcaattac
KLF4	cgggaagggagaagacact	gagttcctcacgccaacg
